# Objective Sleep Duration and All-Cause Mortality Among People With Obstructive Sleep Apnea

**DOI:** 10.1001/jamanetworkopen.2023.46085

**Published:** 2023-12-05

**Authors:** Yiqi Lin, Yongxi Wu, Qianwen Lin, Yun Kwok Wing, Lili Xu, Junbo Ge, Qinwei Wu, Zhen Li, Qingjie Wu, Beiwei Lin, Shichao Wei

**Affiliations:** 1 Department of Sleep Center, Fujian Provincial Hospital, Shengli Clinical Medical College, Fujian Medical University, Fuzhou, China; 2 Department of Psychiatry, Faculty of Medicine, The Chinese University of Hong Kong, Hong Kong SAR, China; 3 Li Chiu Kong Family Sleep Assessment Unit, Department of Psychiatry, Faculty of Medicine, The Chinese University of Hong Kong, Hong Kong SAR, China; 4 Department of Cardiology, Zhongshan Hospital, Fudan University, Shanghai Institute of Cardiovascular Diseases, National Clinical Research Center for Interventional Medicine, China

## Abstract

**Importance:**

The association between sleep duration and all-cause mortality remains unclear among people with obstructive sleep apnea (OSA).

**Objective:**

To explore whether there is an association between sleep duration and all-cause mortality among people with OSA.

**Design, Setting, and Participants:**

This cohort study investigated participants with OSA from the Sleep Heart Health Study (SHHS) in which participants were enrolled between 1995 and 1998 with questionnaires and polysomnography (PSG) assessment and followed up for a median of 11.8 years. SHHS was a multicenter community-based study; 2574 participants with OSA defined by apnea-hypopnea index (AHI) greater than or equal to 15 from SHHS were found; all of them had all-cause mortality data and were included in the study. Data were analyzed from November 2022 to October 2023.

**Exposures:**

Participants were divided into 4 groups with objective sleep duration of (1) at least 7 hours, (2) 6 to less than 7 hours, (3) 5 to less than 6 hours, and (4) less than 5 hours, which was determined by total sleep time on PSG at baseline.

**Main Outcomes and Measures:**

All-cause mortality was defined as deaths from any cause and its risk was compared among 4 OSA groups using Cox regression models.

**Results:**

A total of 2574 participants with OSA were included (1628 [63.2%] men and 946 [36.8%] women; mean [SD] age, 65.4 [10.7] years; 211 [8.2%] Black, 2230 [86.6%] White, 133 [5.2%] other race). Overall, 688 all-cause deaths were observed in participants. Compared with the group sleeping at least 7 hours, the groups sleeping 6 to less than 7 hours (hazard ratio [HR], 1.53 [95% CI, 1.13-2.07]), 5 to less than 6 hours (HR, 1.40 [95% CI, 1.03-1.90]), and less than 5 hours (HR, 1.64 [95% CI, 1.20-2.24]) had significantly higher risks of all-cause mortality independent of AHI. Sensitivity analyses were performed among participants with available data of positive airway pressure treatment during follow-up and the finding was mostly consistent, albeit the HR for the group of 5 to less than 6 hours was not statistically significant.

**Conclusions and Relevance:**

In this cohort study of 2574 participants with OSA, those with shorter objective sleep duration had higher risk of all-cause mortality independent of AHI compared with those sleeping at least 7 hours. Further studies would be needed to investigate health benefits of extending sleep length among people with OSA with short sleep duration.

## Key Points

**Question:**

Is there an association between sleep duration and all-cause mortality among people with obstructive sleep apnea?

**Findings:**

In this cohort study of 2574 participants with obstructive sleep apnea from the Sleep Heart Health Study, compared with participants with objective sleep duration of at least 7 hours, those sleeping less than 7 hours had significantly higher risks of all-cause mortality independent of apnea-hypopnea index.

**Meaning:**

These results suggest that further studies are needed to investigate health benefits of extending sleep length among people with obstructive sleep apnea and short sleep duration.

## Teaser

This cohort study investigates whether there is an association between sleep duration and all-cause mortality among people with obstructive sleep apnea.

## Introduction

Obstructive sleep apnea (OSA) is a common sleep disorder with multiple adverse health outcomes, including cardiovascular disease and cognitive decline.^1,2,3,4^ Increasing studies showed significantly higher risk of all-cause mortality among people with moderate-severe^5^ or severe OSA.^6,7^ However, a traditionally used assessment of severity of OSA, apnea-hypopnea index (AHI), was not always found to predict mortality in OSA patients.^8,9^ Intriguingly, no significant benefit of continuous positive airway pressure (CPAP) was reported on cardiovascular prognosis among people with moderate-severe OSA in randomized clinical trials.^10,11,12^ Therefore, other measurements in addition to AHI would be needed to identify people with OSA at high risk of poor prognosis.^13^

Sleep duration is the most important biomarker of sleep quantity and has effects on various systems.^14,15,16^ Short sleep duration was reported to be associated with multiple cardiovascular and endocrine diseases^16,17,18,19^ as well as all-cause mortality in the general population.^20,21,22^ In recent years, short sleep duration in people with OSA was found to be associated with insulin resistance, visceral obesity, and hypertension.^23,24,25^ Nevertheless, the association between sleep duration and all-cause mortality in OSA was rarely investigated. One retrospective study found that suspected people with OSA with objective sleep duration of 4.9 hours had significantly higher risk of composite cardiovascular outcomes (cardiovascular events and all-cause mortality) compared with those sleeping 6.4 hours but 21% of the patients had AHI less than 5.^8^ Therefore, the association between sleep duration and all-cause mortality remains unclear in people with OSA. To address this question, our study analyzed data of participants with OSA from the Sleep Heart Health Study (SHHS)^26^ and compared all-cause mortality risk in the groups of different objective or subjective sleep duration so as to find out the association between sleep duration and all-cause mortality in OSA.

## Methods

### Study Design and Participants

SHHS is a multicenter prospective community-based cohort study.^26^ A total of 6441 participants older than 40 years were enrolled between 1995 and 1998 from community studies. Participants treated by CPAP, dental device, tracheostomy, and home oxygen therapy were excluded at baseline in SHHS. Participants assessed by questionnaires and home-based polysomnography (PSG) recordings were followed up for a median of 11.8 years. There were available data on 5804 individuals (participants of the Strong Heart Study were not included in the shared SHHS data) at the National Sleep Research Resource (accessed in November 2022).^27,28^ The inclusion criteria of our study was participants with (1) OSA defined by AHI of at least 15 considering substantial disease burden of moderate-severe OSA^29,30^ (AHI was determined as the number of apneas plus hypopneas [with ≥30% nasal cannula or alternative sensor reduction and ≥3% oxygen desaturation or with arousal] per hour of sleep); and (2) all-cause mortality data. Finally, 2574 participants with OSA were found in SHHS, and they all had all-cause mortality data. Therefore, they were all included in our study. The SHHS was conducted in accordance with the Declaration of Helsinki. The institutional review board of each participating institution approved the study protocol and all the participants provided informed written consent. This study followed the Strengthening the Reporting of Observational Studies in Epidemiology (STROBE) reporting guideline.^31^

### Mortality Outcome and Exposure Assessment

Deaths from any cause were identified and confirmed by multiple approaches including follow-up interviews, written annual questionnaires or telephone contacts with study participants or next-of-kin, surveillance of local hospital records and community obituaries, and linkage with the Social Security Administration Death Master File.^7^Objective sleep duration was defined by total sleep time on electroencephalogram-based PSG recordings (Compumedics PSeries System) at home during 1 night which was scored using Rechtschaffen and Kales criteria by trained technicians at a centralized reading center.^32^ Sleep duration ranging from 7 to 9 hours was considered to be normal.^33^ As none of the participants with OSA had objective sleep duration greater than 9 hours (8.6 hours at maximum), participants were divided into 4 groups according to the following sleep duration levels: (1) at least 7 hours, (2) 6 to less than 7 hours, (3) 5 to less than 6 hours, and (4) less than 5 hours.

Additionally, habitual subjective sleep duration on weekdays was determined by the following question: “How many hours of sleep do you usually get at night (or your main sleep period) on weekdays or workdays?” Habitual subjective sleep duration on weekends was determined by this question: “How many hours of sleep do you usually get at night (or your main sleep period) on weekends or your non-workdays?” Habitual sleep duration was calculated by the result of (habitual sleep duration on weekday × 5 + habitual sleep duration on weekends × 2) / 7.^34,35^ Participants with different habitual sleep duration (greater than 9 hours, 7 to 9 hours, 6 to less than 7 hours, 5 to less than 6 hours, and less than 5 hours) were also compared in survival analysis.

### Covariates Assessment

#### Sociodemographic Characteristics and Medical History at Baseline

Sociodemographic characteristics and lifestyle factors at baseline were investigated including age, self-reported gender (men and women), self-reported race (Black, White, other [which included American Indian or Alaska Native, Asian, Hispanic, Native Hawaiian or Other Pacific Islander, multiracial, and others]), body mass index (BMI), and history of ever smoking. Race was assessed because it may have potential effects on the association between sleep duration and all-cause mortality. Medical history was analyzed including hypertension, diabetes, cardiovascular disease, and chronic obstructive pulmonary disease (COPD). Hypertension was based on second and third blood pressure measurements or use of antihypertensive medications.^35^ Diabetes was determined by self-reported history or use of insulin or hypoglycemic medications.^34^ Cardiovascular disease (CVD) history was referred to the parent study cohorts or provided by self-report on the basis of physician-reported angina, myocardial infarction, heart failure or stroke, or history of coronary revascularization procedures. Also, medication history was determined within 2 weeks at baseline including lipid-lowering medication, benzodiazepines, antidepressants (tricyclic antidepressants and nontricyclic antidepressants other than monoamine oxidase inhibitor).

### Other Sleep Assessment at Baseline

Self-reported sleep duration on PSG nights was investigated by the question of “How long did you sleep last night?” after participants got up in the morning following PSG. Sleep perception was determined as the ratio of self-reported sleep duration on PSG nights by total sleep time on PSG.^36^ Epworth Sleepiness Scale (ESS) was used to assess daytime sleepiness and participants with an ESS score greater than or equal to 10 were considered to have excessive daytime sleepiness (EDS).^37^ Insomnia symptoms were assessed by the following questionnaire items: “Have trouble falling asleep,” “Wake up during the night and have difficulty getting back to sleep,” “Wake up too early in the morning and be unable to get back to sleep,” or “Take sleeping pills or other medication to help you sleep.” Participants were considered to have insomnia symptoms if their frequency was 16 to 30 nights per month in at least 1 item.^34^ Apart from total sleep time, other PSG metrics were also analyzed including sleep efficiency, sleep onset latency, wake after sleep onset (WASO), arousal index, and the percentage of total sleep time with oxyhemoglobin saturation below 90%.

### CPAP Treatment and Objective Sleep Duration During Follow-Up

Two to 3 years after the baseline assessment, the participants were followed up with an interim telephone call or clinic visit and were asked if they were treated by positive airway pressure (PAP) since baseline. Participants with PAP treatment were those who responded yes to (1) ever-snoring history and having treatment for snoring with prescription of PAP or (2) being diagnosed with sleep apnea and having treatment for sleep apnea with prescription of PAP. However, PAP treatment duration and adherence were unknown. After approximately 5 years after baseline, PSG was repeated among surviving participants who agreed and were not treated by CPAP, oral device, oxygen therapy, or open tracheostomy in the past 3 months. The change of objective sleep duration was calculated as duration at follow-up minus duration at baseline.

### Statistical Analysis

Statistical analysis was performed by Stata 15/SE (StataCorp) and R version 4.3.1 (R Project for Statistical Computing) from November 2022 to October 2023. Baseline characteristics were compared between 4 OSA groups using Pearson χ^2^ tests for categorical variables and analysis of variance for continuous variables. Logistic regression was used to analyze the association of objective sleep duration with medical history at baseline. Spearman correlation was used to analyze the association of objective sleep duration at baseline with habitual sleep duration and other PSG metrics.

Survival time was defined from date of baseline PSG until date of death or date of censoring. Censoring time was the last time the patient was known to be alive. The crude probability of mortality over time was investigated by Kaplan-Meier survival estimates. Cox regression models were used to analyze all-cause mortality risks in OSA groups by hazard ratios (HRs) and 95% CIs. Adjusted models were constructed to adjust different combinations of potential confounding covariates. The interactions between groups and age or gender were analyzed in the fully adjusted models and proportional hazards assumptions were tested by Schoenfeld residuals. Performed with package plotRCS in R, the restricted cubic spline with 4 knots at the 5th, 35th, 65th, and 95th centiles was used to flexibly model the association of objective sleep duration and all-cause mortality. Pairwise comparison in Cox regression models were performed by using each sleep duration group as the reference group. Sensitivity analysis was performed among participants with available PAP treatment data, and also after excluding those with death within 2 years of baseline or receiving benzodiazepines within 2 weeks at baseline. Overall statistical significance level was set at 2-sided *P* < .05.

## Results

### Baseline Assessment

Among a total of 2574 participants with OSA included in the study, 1628 (63.2%) were men, 946 (36.8%) were women, 211 (8.2%) were Black, 2230 (86.6%) were White, and 133 (5.2%) were other race; and mean (SD) age was 65.4 (10.7) years. There were 341 participants (13.3%) with objective sleep duration of at least 7 hours, 909 (35.3%) with 6 to less than 7 hours, 801 (31.1%) with 5 to less than 6 hours, and 523 (20.3%) with less than 5 hours. In addition, there were 1691 participants (67.2%) who reported habitual sleep duration of 7 to 9 hours, 524 (20.8%) who reported 6 to less than 7 hours, 178 (7.1%) who reported 5 to less than 6 hours, 68 (2.7%) who reported less than 5 hours, and 57 (2.3%) who reported more than 9 hours (56 participants with missing data). In Cox regression analysis, 3 participants who dropped out at the first day were excluded, and there were 2364 and 2316 participants with complete data of covariates in the fully adjusted models for objective and habitual sleep duration analysis, respectively (eTable 1 in Supplement 1).

Among 4 groups with different objective sleep duration, significant differences were found in age, gender, race, smoking history, hypertension, CVD, and insomnia comorbidities. Significant differences were also found in habitual sleep duration, self-reported sleep duration on PSG nights and sleep perception, WASO, arousal index, and other PSG metrics but not AHI (Table 1). Objective sleep duration was found to be significantly associated with habitual sleep duration, sleep perception, and other PSG metrics including AHI, WASO, and arousal index (eTable 2 in Supplement 1). In logistic regression analysis, objective sleep duration less than 5 hours was significantly associated with hypertension history with reference to sleep duration of at least 7 hours at baseline (odds ratio [OR], 1.56 [95% CI, 1.17-2.07]; *P* = .003) after adjustment of age, gender, and race, but objective sleep duration was not significantly associated with CVD, diabetes, and COPD history.

**Table 1.** Demographic and Clinical Characteristics of Participants With OSA With Different Objective Sleep Duration at Baseline

Characteristic	Participants, No. (%)				*P* value
	Objective sleep duration				
	≥7 h (n = 341)	6 to <7 h (n = 909)	5 to <6 h (n = 801)	<5 h (n = 523)	
Sociodemographics					
Age, mean (SD), y	63.1 (11.3)	64.7 (10.5)	65.6 (10.4)	67.8 (10.6)	<.001
BMI, mean (SD)	29.2 (5.1)	29.6 (5.1)	29.5 (5.3)	29.8 (5.7)	.48
Gender					
Women	169 (49.6)	337 (37.1)	246 (30.7)	194 (37.1)	<.001
Men	172 (50.4)	572 (62.9)	555 (69.3)	329 (62.9)	
Race					
Black	20 (5.9)	56 (6.2)	66 (8.2)	69 (13.2)	<.001
White	299 (87.7)	800 (88.0)	698 (87.1)	433 (82.8)	
Other^a^	22 (6.5)	53 (5.8)	37 (4.6)	21 (4.0)	
History of ever smoking	167 (49.4)	493 (54.2)	447 (56.3)	321 (62.0)	.002
Medical history					
Hypertension	148 (43.4)	418 (46.0)	386 (48.2)	311 (59.5)	<.001
Diabetes	33 (10.1)	86 (9.8)	81 (10.6)	66 (13.1)	.28
CVD	72 (21.6)	180 (20.5)	166 (21.7)	146 (30.0)	.001
COPD	1 (0.3)	6 (0.7)	7 (0.9)	10 (2.0)	.05
Lipid-lowering medication	49 (14.5)	120 (13.2)	104 (13.1)	82 (15.8)	.49
Antidepressants usage	28 (8.3)	56 (6.2)	44 (5.5)	30 (5.8)	.35
Benzodiazepines usage	10 (3.0)	30 (3.3)	32 (4.0)	25 (4.8)	.42
Sleep-related covariates					
Insomnia comorbidity	45 (13.5)	97 (10.9)	114 (14.5)	82 (16.0)	.03
EDS comorbidity	118 (34.9)	342 (38.0)	307 (38.6)	196 (38.4)	.68
Habitual sleep duration on weekdays, mean (SD), h	7.4 (1.2)	7.1 (1.1)	6.9 (1.2)	6.8 (1.3)	<.001
Habitual sleep duration on weekends, mean (SD), h	7.7 (1.3)	7.5 (1.2)	7.3 (1.3)	7.1 (1.4)	<.001
Habitual sleep duration, mean (SD), h	7.5 (1.2)	7.2 (1.1)	7.1 (1.2)	6.9 (1.3)	<.001
PSG results					
TST, mean (SD), min	444.1 (18.8)	387.8 (16.5)	331.6 (17.0)	253.6 (41.4)	<.001
Self-reported sleep duration on PSG nights, mean (SD), min	461.2 (80.9)	417.1 (73.4)	379.4 (77.7)	343.5 (92.7)	<.001
Sleep perception, mean (SD), %	103.9 (17.8)	107.6 (18.8)	114.6 (23.7)	139.3 (49.3)	<.001
WASO, mean (SD), min	42.6 (20.5)	57.6 (31.5)	74.2 (42.2)	101.8 (66.8)	<.001
Sleep efficiency, mean (SD), %	89.9 (4.1)	85.2 (6.2)	79.6 (8.5)	69.3 (14.1)	<.001
Sleep onset latency, mean (SD), min	7.8 (12.0)	11.7 (15.6)	15.4 (21.0)	20.0 (27.0)	<.001
Arousal index, mean (SD), events/h	21.6 (11.6)	24.0 (11.1)	24.7 (11.8)	27.0 (14.1)	<.001
N1 percentage, mean (SD), %	4.8 (3.2)	5.8 (3.8)	6.4 (4.4)	7.6 (6.0)	<.001
N2 percentage, mean (SD), %	58.6 (10.9)	58.8 (10.8)	59.2 (11.5)	58.4 (13.0)	.62
N3 percentage, mean (SD), %	16.2 (10.8)	15.8 (11.1)	15.8 (11.7)	17.1 (12.9)	.19
REM percentage, mean (SD), %	20.4 (5.4)	19.7 (5.4)	18.6 (6.2)	16.9 (7.8)	<.001
AHI, mean (SD), events/h	29.6 (16.5)	30.6 (15.5)	31.2 (15.7)	32.4 (17.3)	.07
TST90, mean (SD), %	5.5 (13.2)	5.8 (12.4)	6.4 (12.9)	7.4 (14.2)	.10
Outcomes					
All-cause mortality	58 (17.0)	240 (26.4)	209 (26.1)	181 (34.6)	<.001

Abbreviations: AHI, apnea-hypopnea index; BMI, body mass index (calculated as weight in kilograms divided by height in meters squared); COPD, chronic obstructive pulmonary disease; CVD, cardiovascular disease; EDS, excessive daytime sleepiness; N1, non–rapid eye movement sleep stage 1; N2, non–rapid eye movement sleep stage 2; N3, non–rapid eye movement sleep stage 3; OSA, obstructive sleep apnea; PSG, polysomnography; REM, rapid eye movement; TST, total sleep time; TST90, total sleep time with oxyhemoglobin saturation below 90%; WASO, wake after sleep onset.

^a^ The other race category included American Indian or Alaska Native, Asian, Hispanic, Native Hawaiian or Other Pacific Islander, multiracial, and others.

### CPAP Treatment and Objective Sleep Duration During Follow-Up

PAP treatment data was available for 2195 participants during follow-up. Among them, 94 participants (4.3%) were treated by PAP and no difference was found among 4 objective sleep duration groups (≥7 hours: 3.0% [9 of 298]; 6 to <7 hours: 5.6% [43 of 774]; 5 to <6 hours: 4.0% [27 of 684]; <5 hours: 3.4% [15 of 439]; *P* = .16). Among 1135 OSA participants who repeated PSG, the mean (SD) change in objective sleep duration was 0.1 (1.3) hours. A significant correlation was found between objective sleep duration at baseline and follow-up (Spearman correlation coefficient, 0.33 [95% CI, 0.28-0.39]). Notably, the participants repeating PSG were younger and with lower AHI and longer objective sleep duration when compared with those not repeating PSG at baseline (eTable 3 in Supplement 1).

### Mortality

**Figure 1.**
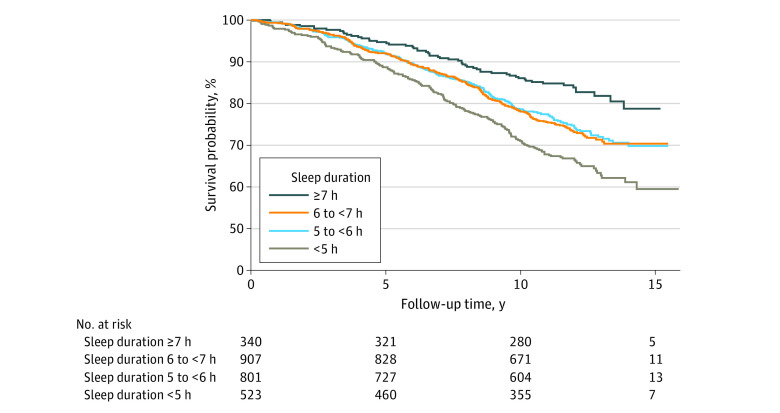
Unadjusted Kaplan-Meier Curves Across Groups With Different Objective Sleep Duration for All-Cause Mortality

The median (IQR) follow-up period for all-cause mortality among our study’s participants was 11.7 (9.8-12.6) years. Among a total of 688 deaths observed among participants, 58 (17.0%) were in the group with sleep duration of at least 7 hours, 240 (26.4%) with 6 to less than 7 hours, 209 (26.1%) with 5 to less than 6 hours, and 181 (34.6%) with less than 5 hours (*P* < .001) (Table 1). Kaplan-Meier curves were shown in Figure 1.

**Figure 2.**
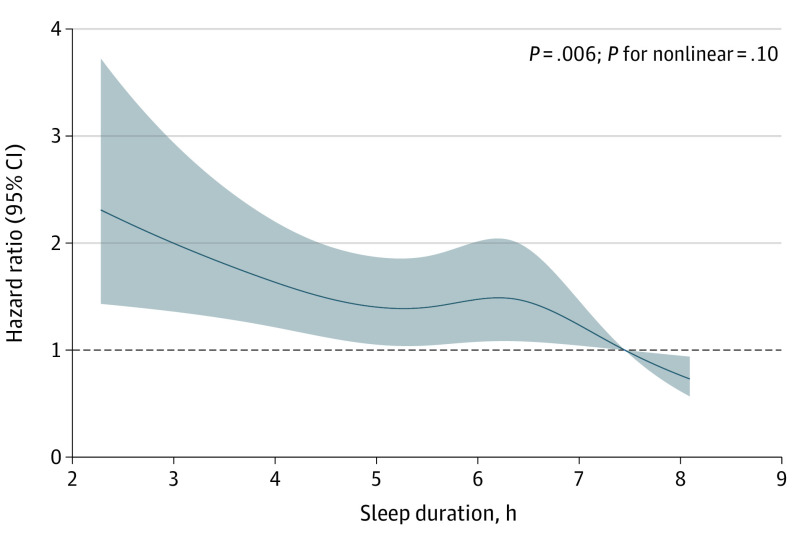
Restricted Cubic Spline of Association Between Objective Sleep Duration and All-Cause Mortality The association was adjusted for age; gender; race; smoking history; body mass index; history of diabetes, cardiovascular disease, hypertension, chronic obstructive pulmonary disease; usage of lipid-lowering medication and antidepressants within 2 weeks at baseline; and apnea-hypopnea index. The 95th percentile was used as the reference value. Shaded areas indicate 95% CIs.

Compared with the group sleeping at least 7 hours, the other 3 groups had significantly higher risks of all-cause mortality after adjustment for age, gender, race, smoking history, BMI, history of diabetes, CVD, hypertension and COPD, usage of lipid-lowering medication and antidepressants, and AHI (6 to <7 hours: HR, 1.53 [95% CI, 1.13-2.07]; 5 to <6 hours: HR, 1.40 [95% CI, 1.03-1.90]; <5 hours: HR, 1.64 [95% CI, 1.20-2.24]) (Table 2). No significant interactions were found between groups and age or gender in the fully adjusted model. Nevertheless, no significant association was found between habitual sleep duration and all-cause mortality (Table 3). The restricted cubic spline was then used to visualize nonlinearity and the plot showed a seemingly inverted U-shape curve around objective sleep duration of 5 to 7 hours but the nonlinearity test suggested it was not statistically significant (Figure 2). Pairwise comparisons were further performed in the fully adjusted Cox regression model and no significant difference was found in the risk of all-cause mortality between groups with objective sleep duration of 6 to less than 7 hours, 5 to less than 6 hours, and less than 5 hours (eTable 4 in Supplement 1).

**Table 2.** Hazard Ratios of All-Cause Mortality Among Participants With Obstructive Sleep Apnea With Different Objective Sleep Duration

Model	Participants, No.	Events, No.	Hazard ratio (95% CI)			
			≥7 h	6 to <7 h	5 to <6 h	<5 h
1^a^	2571	688	1 [Reference]	1.65 (1.24-2.20)	1.60 (1.20-2.14)	2.27 (1.69-3.04)
2^b^	2571	688	1 [Reference]	1.52 (1.14-2.03)	1.33 (0.99-1.78)	1.64 (1.21-2.21)
3^c^	2364	643	1 [Reference]	1.54 (1.14-2.07)	1.41 (1.04-1.91)	1.64 (1.20-2.24)
4^d^	2364	643	1 [Reference]	1.53 (1.13-2.07)	1.40 (1.03-1.90)	1.64 (1.20-2.24)

^a^ Model 1: without adjustment.

^b^ Model 2: after adjustment for age, gender, and race.

^c^ Model 3: after adjustment for covariates in model 2 plus smoking history, body mass index, history of diabetes, cardiovascular disease, hypertension and chronic obstructive pulmonary disease, and usage of lipid-lowering medication and antidepressants within 2 weeks at baseline.

^d^ Model 4: after adjustment for covariates in model 3 plus apnea-hypopnea index.

**Table 3.** Hazard Ratios of All-Cause Mortality Among Participants With Obstructive Sleep Apnea With Different Habitual Sleep Duration

Model	Participants, No.	Events, No.	Hazard ratio (95% CI)				
			7 to 9 h	6 to <7 h	5 to <6 h	<5 h	>9 h
1^a^	2515	666	1 [Reference]	0.93 (0.76-1.13)	1.18 (0.89-1.57)	1.38 (0.91-2.10)	1.59 (1.02-2.46)
2^b^	2515	666	1 [Reference]	1.01 (0.83-1.23)	1.19 (0.89-1.60)	1.31 (0.86-2.00)	1.06 (0.68-1.64)
3^c^	2316	623	1 [Reference]	1.00 (0.82-1.23)	1.05 (0.77-1.43)	1.07 (0.69-1.64)	1.05 (0.67-1.63)
4^d^	2316	623	1 [Reference]	1.01 (0.82-1.23)	1.05 (0.77-1.42)	1.07 (0.69-1.64)	1.05 (0.67-1.64)

^a^ Model 1: without adjustment.

^b^ Model 2: after adjustment for age, gender, and race.

^c^ Model 3: after adjustment for covariates in model 2 plus smoking history, body mass index, history of diabetes, cardiovascular disease, hypertension and chronic obstructive pulmonary diseasec, and usage of lipid-lowering medication and antidepressants within 2 weeks at baseline.

^d^ Model 4: after adjustment for covariates in model 3 plus apnea-hypopnea index.

Among participants who repeated PSG measures, the results showed that within each objective sleep duration group, compared with participants with unchanged sleep duration levels, similar risk of all-cause mortality was found in participants with different sleep duration trajectories in most cases. Among participants whose sleep duration of 5 to less than 6 hours at baseline decreased to less than 5 hours during follow-up, a slightly higher risk of all-cause mortality was found compared with those with persistent sleep duration of 5 to less than 6 hours (eTable 5 in Supplement 1).

After excluding participants who died within 2 years of baseline or were receiving benzodiazepines within 2 weeks at baseline assessments, the risks remained significantly higher in participants with objective sleep duration of 6 to less than 7 hours, 5 to less than 6 hours, and less than 5 hours compared with those sleeping at least 7 hours (eTables 6 and 7 in Supplement 1). Among participants with available PAP treatment data, the finding was mostly consistent, albeit not statistically significant in the group of 5 to less than 6 hours (eTable 8 in Supplement 1).

## Discussion

Our study reported that compared with participants with OSA with objective sleep duration of at least 7 hours, those with shorter sleep duration were at higher risk of all-cause mortality independent of AHI. This finding was mostly consistent among participants with available CPAP treatment data during follow-up. Interestingly, no significant association was found between all-cause mortality and self-reported habitual sleep duration in OSA.

Objective sleep duration less than 7 hours accounted for 86.7% of our study’s participants, and the percentage was seemingly higher than that of another OSA study (37.9%),^25^ which additionally included mild OSA with AHI 5 to less than 15 and excluded participants with sleep-disrupting conditions or other sleep disorders. Also, only 30.6% of our OSA participants reported their habitual sleep duration as less than 7 hours. The discrepancy between objective and habitual sleep duration could be partly explained by sleep misperception in OSA^38,39,40^ and the first night effect resulting in shorter objective sleep duration, albeit home-based PSG was shown to report similar sleep duration across consecutive nights among patients with OSA.^41^

Our study found that participants with OSA and objective sleep duration of 6 to less than 7 hours, 5 to less than 6 hours, and less than 5 hours were at higher risk of all-cause mortality independent of AHI compared with participants with OSA and sleep duration of at least 7 hours. This association remained significant even after excluding those participants who died within 2 years of baseline and reported usage of benzodiazepines. During follow-up at 5 years, the change of sleep duration was minimal and did not affect the risk of all-cause mortality in most cases, albeit the results should be interpreted with caution as participants repeating PSG were younger and with lower AHI and longer objective sleep duration at baseline. Consistently, lower risk of composite cardiovascular outcomes was previously reported in suspected cases of OSA among patients sleeping 6.4 hours compared with those sleeping 4.9 hours.^8^ Short sleep duration in OSA was found to be significantly associated with hypertension history at baseline in our study and 1 previous PSG study.^25^ Short sleep duration was also reported to be associated with other adverse health outcomes in OSA, including altered insulin resistance^23^ and visceral obesity.^24^

The underlying mechanism remains unclear. Among our study’s participants with OSA, significant negative association was found between objective sleep duration and WASO and arousal index. Sleep fragmentation was considered to play a major role in most consequences of OSA^42^ and arousal burden was found to be associated with all-cause mortality in older adults.^43^ Therefore, sleep fragmentation with frequent arousals may contribute to the association between all-cause mortality and sleep duration in OSA. Also, short sleep duration could affect inflammation and hormone metabolism^20,21^ and was associated with elevated myeloperoxidase levels in OSA,^44^ suggesting its possible role in increasing oxidative stress and altered inflammation pathways in OSA.

Unlike objective sleep duration, subjective habitual sleep duration was not found to be associated with all-cause mortality in OSA. Similarly, objective but not subjective sleep duration was reported to be associated with hypertension in patients with OSA^25^ and patients with insomnia.^45^ These discrepancies may be partly explained by sleep misperception in OSA.^38,39,40^ In our study, the correlation between objective and habitual sleep duration was significant but weak. Therefore, objective measurement of habitual sleep duration would be warranted, such as with ambulatory PSG on consecutive nights in the future.

PAP treatment was only reported in 94 (4.3%) of our OSA participants who had follow-up data, which limited its influence on overall results. In view of benefits of PAP treatment on all-cause mortality reported in SHHS,^46^ we also compared the usage percentage of PAP among different objective sleep duration groups and did not find any difference. Further sensitivity analysis was performed among those with available PAP treatment data and the finding was mostly consistent.

### Limitations

Our study had limitations. First, there may be reduction of objective sleep duration due to first night effect in our 1-night study, although it was previously found to be insignificant in home-based PSG of people with OSA.^41^ Second, habitual sleep duration was investigated on the basis of self-reports and in the future, ambulatory PSG on consecutive nights would be warranted. Third, as none of our participants had objective sleep duration greater than 9 hours at baseline, mortality of participants with long sleep duration was only investigated based on subjective but not objective measurement. Fourth, only 44.1% of participants repeated PSG at 5 years with lower AHI and longer objective sleep duration compared with those without repeated PSG. Fifth, there may be potential selection bias as participants with CPAP treatment were excluded at baseline. Additionally, during follow-up at 2 to 3 years, not all (85.3%) had available PAP treatment data with their treatment duration unknown. Their long-term PAP adherence was also unclear. Nevertheless, further sensitivity analysis was performed and the finding was mostly consistent.

## Conclusions

In this cohort study of participants with OSA with a median of 11.7 years of follow-up, participants with shorter objective sleep duration were at higher risk of all-cause mortality compared with those with sleep duration of at least 7 hours. Further research would be needed to shed light on its underlying mechanism and possible health benefits of extending sleep length among people with OSA with short sleep duration by sleep education or other sleep intervention.

## Notes

Supplement 1. **eTable 1.** Number of Participants With Missing Data of Each Covariate in Fully Adjusted Models of Cox Regression **eTable 2.** Spearman’s Correlation of Objective Sleep Duration With Other Sleep Metrics at Baseline **eTable 3.** Comparison of Clinical Characteristics Between Participants With and Without Available PAP Treatment Data and Between Participants With and Without Repeated PSG **eTable 4.** Pairwise Comparisons Between Different Sleep Duration Groups in the Fully Adjusted Cox Regression Model **eTable 5.** Hazard Ratios (95% Confidence Interval) of All-Cause Mortality in Different Trajectories of Objective Sleep Duration **eTable 6.** Hazard Ratios (95% Confidence Interval) of All-Cause Mortality in OSA Participants by Excluding Those Who Died Within Two Years of Baseline **eTable 7.** Hazard Ratios (95% Confidence Interval) of All-Cause Mortality in OSA Participants by Excluding Those Who Were Taking Benzodiazepines Within Two Weeks at Baseline **eTable 8.** Hazard Ratios (95% Confidence Interval) of All-Cause Mortality in Different Objective Sleep Duration Groups Among OSA Participants With Available PAP Treatment Data

Supplement 2.
Data Sharing Statement
