# Supplementary material for: Objective Sleep Duration and All-Cause Mortality Among People With Obstructive Sleep Apnea
Source: JAMA Netw Open. 2023 Dec 5;6(12):e2346085. doi: 10.1001/jamanetworkopen.2023.46085 (PMC10698624; doi:10.1001/jamanetworkopen.2023.46085)
Supplement: Supplement 1. — eTable 1. Number of Participants With Missing Data of Each Covariate in Fully Adjusted Models of Cox Regression eTable 2. Spearman’s Correlation of Objective Sleep Duration With Other Sleep Metrics at Baseline eTable 3. Comparison of Clinical Characteristics Between Participants With and Without Available PAP Treatment Data and Between Participants With and Without Repeated PSG eTable 4. Pairwise Comparisons Between Different Sleep Duration Groups in the Fully Adjusted Cox Regression Model eTable 5. Hazard Ratios (95% Confidence Interval) of All-Cause Mortality in Different Trajectories of Objective Sleep Duration eTable 6. Hazard Ratios (95% Confidence Interval) of All-Cause Mortality in OSA Participants by Excluding Those Who Died Within Two Years of Baseline eTable 7. Hazard Ratios (95% Confidence Interval) of All-Cause Mortality in OSA Participants by Excluding Those Who Were Taking Benzodiazepines Within Two Weeks at Baseline eTable 8. Hazard Ratios (95% Confidence Interval) of All-Cause Mortality in Different Objective Sleep Duration Groups Among OSA Participants With Available PAP Treatment Data [file jamanetwopen-e2346085-s001.pdf]

Supplemental Online Content

Lin Y, Wu Y, Lin Q, et al. Objective sleep duration and all-cause mortality among people with obstructive sleep apnea. *JAMA Netw Open.* 2023;6(12):e2346085. doi:10.1001/jamanetworkopen.2023.46085

eTable 1. Number of Participants With Missing Data of Each Covariate in Fully Adjusted Models of Cox Regression

eTable 2. Spearman’s Correlation of Objective Sleep Duration With Other Sleep Metrics at Baseline

eTable 3. Comparison of Clinical Characteristics Between Participants With and Without Available PAP Treatment Data and Between Participants With and Without Repeated PSG

eTable 4. Pairwise Comparisons Between Different Sleep Duration Groups in the Fully Adjusted Cox Regression Model

eTable 5. Hazard Ratios (95% Confidence Interval) of All-Cause Mortality in Different Trajectories of Objective Sleep Duration

eTable 6. Hazard Ratios (95% Confidence Interval) of All-Cause Mortality in OSA Participants by Excluding Those Who Died Within Two Years of Baseline

eTable 7. Hazard Ratios (95% Confidence Interval) of All-Cause Mortality in OSA Participants by Excluding Those Who Were Taking Benzodiazepines Within Two Weeks at Baseline

eTable 8. Hazard Ratios (95% Confidence Interval) of All-Cause Mortality in Different Objective Sleep Duration Groups Among OSA Participants with Available PAP Treatment Data

This supplemental material has been provided by the authors to give readers additional information about their work.

© 2023 Lin Y et al. *JAMA Network Open.*

eTable 1. Number of participants with missing data of covariates in the fully adjusted models of Cox regression

	**number of participants with missing data in each covariate**
For analysis of objective sleep duration and all-cause mortality in OSA participants (Table 2)	smoking history (n=15), BMI (n=10), diabetes (n=102), CVD (n=112), COPD (n=45), history of lipid lowering medication (n=13), history of antidepressants (n=13)
For analysis of habitual sleep duration and all-cause mortality in OSA participants (Table 3)	smoking history (n=14), BMI (n=10), diabetes (n=99), CVD (n=104), COPD (n=42), history of lipid lowering medication (n=12), history of antidepressants (n=12)

BMI: body mass index; COPD: chronic obstructive pulmonary disease; CVD: cardiovascular disease; OSA: obstructive sleep apnea; PSG: polysomnography.

© 2023 Lin Y et al. *JAMA Network Open.*

eTable 2. Spearman's correlation of objective sleep duration with other sleep metrics at baseline

	correlation coefficient (95% confidence interval)	p
Habitual TST	0.16 (0.12 to 0.20)	<.001
Sleep perception	-0.36 (-0.39 to -0.32)	<.001
AHI	-0.08 (-0.12 to -0.04)	<.001
Percent of sleep time in apneas	-0.02 (-0.06 to 0.02)	.30
Percent of sleep time in hypopneas	-0.07 (-0.11 to -0.03)	<.001
WASO	-0.35(-0.39 to -0.32)	<.001
Arousal index	-0.14 (-0.17 to -0.10)	<.001
N1 percentage	-0.15 (-0.19 to -0.11)	<.001
N2 percentage	-0.02 (-0.06 to 0.02)	.41
N3 percentage	-0.01 (-0.05 to 0.03)	.66
REM percentage	0.19 (0.15 to 0.23)	<.001
TST90	-0.07 (-0.11 to -0.03)	<.001
Average oxygen saturation during sleep	0.05 (0.01 to 0.09)	.008

AHI: apnea-hypopnea index; N1: non-rapid eye movement sleep stage 1; N2: non-rapid eye movement sleep stage 2; N3: non-rapid eye movement sleep stage 3; OSA: obstructive sleep apnea; REM: rapid eye movement; TST: total sleep time; TST90: total sleep time with oxyhemoglobin saturation below 90%; WASO: wake after sleep onset.

© 2023 Lin Y et al. *JAMA Network Open.*

eTable 3. Comparison of clinical characteristics between participants with and without available PAP treatment data and between participants with and without repeated PSG

	Participants with and without available PAP treatment data			Participants with and without repeated PSG		
	with PAP data	without PAP data	P	with repeated PSG	without repeated PSG	P
n (%)	2195 (85.3)	379 (14.7)		1135 (44.1)	1439 (55.9)	
Age, years	65.4 (10.5)	65.1 (11.8)	.59	64.4 (10.1)	66.1 (11.1)	.001
BMI, kg/m^2	29.5 (5.2)	29.7 (5.7)	.58	29.7 (5.1)	29.5 (5.4)	.28
Gender, n (%)						
Female	818 (37.3)	128 (33.8)	.19	429 (37.8)	517 (35.9)	
Male	1377 (62.7)	251 (66.2)		706 (62.2)	922 (64.1)	.33
Race, n (%)						
Black	161 (7.3)	50 (13.2)	<.001	72 (6.3)	139 (9.7)	
White	1949 (88.8)	281 (74.1)		1014 (89.3)	1216 (84.5)	.001
Other [a]	85 (3.9)	48 (12.7)		49 (4.3)	84 (5.8)	
PSG Sleep duration at baseline, hours	5.9 (1.1)	5.8 (1.1)	.11	6.0 (1.0)	5.7 (1.1)	<.001
AHI at baseline, events/hours	31.1 (16.1)	30.7 (15.8)	.71	29.6 (14.4)	32.1 (17.2)	<.001
Mortality, n (%)	545 (24.8)	143 (37.7)	<.001	175 (15.4)	513 (35.7)	<.001

AHI: apnea-hypopnea index; BMI: body mass index; PAP: continuous positive airway pressure; PSG: polysomnography.

[a] The category of “other race” included American Indian or Alaska Native, Asian, Hispanic, Native Hawaiian or other Pacific Islander, multi-Racial and others.

© 2023 Lin Y et al. *JAMA Network Open.*

eTable 4. Pairwise comparisons between different sleep duration groups in the fully adjusted Cox regression model [a]

Objective sleep duration		Habitual sleep duration	
Pairwise comparison [b]	Hazard ratios (95% CI)	Pairwise comparison [b]	Hazard ratios (95% CI)
6 to <7h vs ≥7h	1.53 (1.13-2.07)	6 to <7h vs 7 to 9h	1.01 (0.82-1.23)
5 to <6h vs ≥7h	1.40 (1.03-1.90)	5 to <6h vs 7 to 9h	1.05 (0.77-1.42)
<5h vs ≥7h	1.64 (1.20-2.24)	<5h vs 7 to 9h	1.07 (0.69-1.64)
5 to <6h vs 6 to <7h	0.92 (0.75-1.11)	>9h vs 7 to 9h	1.05 (0.67-1.64)
<5h vs 6 to <7h	1.07 (0.87-1.31)	5 to <6h vs 6 to <7h	1.04 (0.74-1.46)
<5h vs 5 to <6h	1.17 (0.95-1.44)	<5h vs 6 to <7h	1.06 (0.67-1.67)
		>9h vs 6 to <7h	1.04 (0.65-1.66)
		<5h vs 5 to <6h	1.02 (0.61-1.68)
		>9h vs 5 to <6h	1.00 (0.59-1.68)
		>9h vs <5h	0.98 (0.54-1.80)

CI: confidence interval.

[a] The fully adjusted model was used with covariates of age, gender and race, smoking history, body mass index, history of diabetes, cardiovascular disease, hypertension, chronic obstructive pulmonary disease, usage of lipid lowering medication and antidepressants within 2 weeks at baseline and apnea-hypopnea index.

[b] The reference category is always the second category presented in this column.

© 2023 Lin Y et al. *JAMA Network Open.*

eTable 5. Hazard ratios (95% confidence interval) of all-cause mortality in different trajectories of objective sleep duration

Sleep duration at baseline	Sleep duration during follow-up	Model 1	Model 2	Model 3
≥7h	≥7h		reference	
	6 to <7h	1.65 (0.50-5.49)		
	5 to <6h	2.91 (0.72-11.67)		
	<5h	1.53 (0.17-13.78)		
6 to <7h	6 to <7h		reference	
	≥7h	0.81 (0.40-1.65)	0.94 (0.46-1.93)	
	5 to <6h	1.50 (0.80-2.81)	1.12 (0.59-2.12)	
	<5h	2.53 (1.37-4.69)	1.70 (0.90-3.19)	
5 to <6h	5 to <6h		reference	
	≥7h	0.54 (0.19-1.60)	0.76 (0.26-2.25)	0.88 (0.29-2.66)
	6 to <7h	1.06 (0.54-2.09)	1.47 (0.74-2.92)	1.23 (0.59-2.58)
	<5h	2.48 (1.34-4.59)	2.13 (1.15-3.97)	1.98 (1.02-3.84)
<5h	<5h		reference	
	≥7h	0.12 (0.02-0.87)	0.15 (0.02-1.17)	
	6 to <7h	0.38 (0.14-1.04)	0.80 (0.28-2.33)	
	5 to <6h	0.43 (0.18-1.02)	0.58 (0.24-1.40)	

Model 1: without adjustment; Model 2: after adjustment for age, gender and race; Model 3: after adjustment for covariates in model 2 plus smoking history, body mass index, history of diabetes, cardiovascular disease, hypertension and chronic obstructive pulmonary disease and usage of lipid lowering medication and antidepressants within 2 weeks at baseline and apnea-hypopnea index at baseline.

© 2023 Lin Y et al. *JAMA Network Open.*

eTable 6. Hazard ratios (95% confidence interval) of all-cause mortality in OSA participants by excluding those who died within two years of baseline

model	n	event	≥7h	6 to <7h	5 to <6h	<5h
model 1	2511	628	Ref	1.68 (1.24-2.26)	1.62 (1.19-2.19)	2.24 (1.64-3.05)
model 2	2511	628	Ref	1.54 (1.14-2.09)	1.34 (0.99-1.83)	1.62 (1.19-2.22)
model 3	2310	589	Ref	1.54 (1.13-2.11)	1.39 (1.01-1.91)	1.59 (1.15-2.19)
model 4	2310	589	Ref	1.54 (1.13-2.10)	1.39 (1.01-1.91)	1.59 (1.15-2.19)

OSA: obstructive sleep apnea.

Model 1: without adjustment; Model 2: after adjustment for age, gender, race; Model 3: after adjustment for covariates in model 2 plus smoking history, body mass index, history of diabetes, cardiovascular disease, hypertension and chronic obstructive pulmonary disease, and usage of lipid lowering medication and antidepressants within 2 weeks at baseline; Model 4: after adjustment for covariates in model 3 plus apnea-hypopnea index.

© 2023 Lin Y et al. *JAMA Network Open.*

eTable 7. Hazard ratios (95% confidence interval) of all-cause mortality in OSA participants by excluding those who were taking benzodiazepines within two weeks at baseline

model	n	event	≥7h	6 to <7h	5 to <6h	<5h
model 1	2461	656	Ref	1.64 (1.22-2.19)	1.58 (1.17-2.12)	2.24 (1.66-3.03)
model 2	2461	656	Ref	1.50 (1.11-2.01)	1.29 (0.96-1.75)	1.60 (1.18-2.16)
model 3	2272	614	Ref	1.54 (1.13-2.09)	1.38 (1.01-1.88)	1.62 (1.18-2.22)
model 4	2272	614	Ref	1.54 (1.13-2.09)	1.38 (1.01-1.88)	1.62 (1.18-2.22)

BZDs: benzodiazepines; OSA: obstructive sleep apnea.

Model 1: without adjustment; Model 2: after adjustment for age, gender, race; Model 3: after adjustment for covariates in model 2 plus smoking history, body mass index, history of diabetes, cardiovascular disease, hypertension and chronic obstructive pulmonary disease, and usage of lipid lowering medication and antidepressants within 2 weeks at baseline; Model 4: after adjustment for covariates in model 3 plus apnea-hypopnea index.

© 2023 Lin Y et al. *JAMA Network Open.*

eTable 8. Hazard ratios (95% confidence interval) of all-cause mortality in different objective sleep duration groups among OSA participants with available PAP treatment data

model	n	event	≥7h	6 to <7h	5 to <6h	<5h
model 1	2195	545	Ref	1.66 (1.21-2.29)	1.56 (1.13-2.16)	2.19 (1.57-3.04)
model 2	2195	545	Ref	1.54 (1.12-2.12)	1.33 (0.96-1.85)	1.61 (1.15-2.25)
model 3	2040	512	Ref	1.55 (1.11-2.17)	1.36 (0.97-1.92)	1.57 (1.11-2.23)
model 4	2040	512	Ref	1.55 (1.11-2.16)	1.36 (0.97-1.91)	1.57 (1.11-2.22)

PAP: continuous positive airway pressure; OSA: obstructive sleep apnea; PSG: polysomnography.

Model 1: without adjustment; Model 2: after adjustment for age, gender and race; Model 3: after adjustment for covariates in model 2 plus smoking history, body mass index, history of diabetes, cardiovascular disease, hypertension and chronic obstructive pulmonary disease and usage of lipid lowering medication and antidepressants within 2 weeks at baseline; Model 4: after adjustment for covariates in model 3 plus apnea-hypopnea index.

© 2023 Lin Y et al. *JAMA Network Open.*
