# Supplementary material for: Objective Sleep Duration and All-Cause Mortality Among People With Obstructive Sleep Apnea
Source: JAMA Netw Open. 2023 Dec 5;6(12):e2346085. doi: 10.1001/jamanetworkopen.2023.46085 (PMC10698624; doi:10.1001/jamanetworkopen.2023.46085)
Supplement: Supplement 2. — Data Sharing Statement [file jamanetwopen-e2346085-s002.pdf]

Data Sharing Statement

Lin. Objective Sleep Duration and All-Cause Mortality Among People With Obstructive Sleep Apnea. *JAMA Netw Open*. Published December 05, 2023.
doi:10.1001/jamanetworkopen.2023.46085

Data
Data available: No
